# Neuropharmacological and Antidiarrheal Potentials of *Duabanga grandiflora* (DC.) Walp. Stem Bark and Prospective Ligand–Receptor Interactions of Its Bioactive Lead Molecules

**DOI:** 10.3390/cimb44050159

**Published:** 2022-05-20

**Authors:** Israt Jahan, Mohammad Forhad Khan, Mohammed Abu Sayeed, Laiba Arshad, Md. Amjad Hossen, Md. Jakaria, Duygu Ağagündüz, Md. Areeful Haque, Raffaele Capasso

**Affiliations:** 1 Department of Pharmacy, International Islamic University Chittagong, Chittagong 4318, Bangladesh; jahaanisrat6@gmail.com (I.J.); forhadkhaniiuc@gmail.com (M.F.K.); amjadsajid29@outlook.com (M.A.H.); 2 Department of Pharmacy, Faculty of Life and Earth Sciences, Jagannath University, Dhaka 1100, Bangladesh; 3 Department of Chemistry, University of Massachusetts Boston, Boston, MA 02125, USA; 4 Department of Pharmacy, Forman Christian College (A Chartered University), Lahore 54600, Pakistan; laibaarshad@fccollege.edu.pk; 5 The Florey Institute of Neuroscience and Mental Health, The University of Melbourne, Parkville, VIC 3052, Australia; md.jakaria@florey.edu.au; 6 Department of Nutrition and Dietetics, Faculty of Health Sciences, Gazi University, Emek, Ankara 06490, Turkey; duyguturkozu@gazi.edu.tr; 7 Department of Agricultural Sciences, University of Naples Federico II, 80055 Portici, Italy

**Keywords:** *Duabanga grandiflora* (DC.) Walp., anti-depressant, anxiolytic, anti-diarrheal, vanillin

## Abstract

*Duabanga grandiflora* (DC.) Walp. is an ethnomedicinally significant plant used to treat various illnesses, but there is little scientific evidence to support its use. This study explored the pharmacological activities of methanol extract of *D. grandiflora* stem barks (MEDG) through in vivo approaches in Swiss albino mice and a computer-aided molecular approach. The forced swimming test (FST), tail suspension test (TST), elevated plus maze (EPM), and hole board test (HBT) were used to determine anti-depressant and anxiolytic activity in experimental mice. In addition, anti-diarrheal studies were performed using castor oil-induced diarrhea, castor oil-induced enter pooling, and the charcoal-induced gastrointestinal motility test. MEDG showed substantial depletions in the immobility times in both FST and TST after treatment with the MEDG extract, whereas moderate anxiolytic activity was manifested at a higher dose (400 mg/kg) compared with the control. Correspondingly, MEDG extract revealed a significant reduction in wet feces and decreased the small intestinal transit of charcoal meal in castor oil-induced diarrhea and charcoal-induced gastrointestinal motility test. In the computer-aided molecular approaches, vanillin displayed a promising binding score for both anxiolytic and anti-diarrheal activities, while duabanganal C showed a promising score for the anti-depressant activity. The present experimental findings along with a computer-aided model conclude that MEDG could be a possible Phyto therapeutic agent with potential anti-depressant, anxiolytic and anti-diarrheal activity.

## 1. Introduction

Plant-based natural medicine plays a significant role in the health system of many developed and developing countries, raising their commercial values. Although the use of medicinal plants in the treatment of various disorders has been established for centuries in all parts of the world, the consumption of natural medicines has expanded considerably in recent years [1]. Plant-based herbal medicine and food supplements are gaining popularity around the world because they are less expensive and less toxic than synthetic drugs. Though herbal medicines are not commercially available enough, the development of synthetic drugs based on natural products has increased considerably in recent years. However, secondary metabolites in natural products also play a crucial role in the treatment of different diseases such as neurological disorders, diarrhea, myocardial infarction, stroke, and worm infections [2]. Not only are natural products a great source of therapeutic agents for the last couple of decades, but also a number of modern synthetic drugs have been isolated from natural sources based on their traditional uses.

At present, neurodegenerative disorders are wide spread and, in turn, exert substantial personal, social, and economic stresses. Research-based evidence reveals that reactive oxygen species (ROS) is a pivotal factor in the pathophysiology of certain neurological and psychiatric disorders, including anxiety and depression [3]. Reactive oxygen species development persists throughout the brain protection system that can modify normal neural activity [4]. However, commercially available anti-depressants are widely used for the management of depression even though these drugs have limited success with much more adverse effects. Thus, there is a need for time to develop effective medication with less adverse effects for the management of depressive disorders [5]. Additionally, anxiety seems to be the most widespread neurological disorder, ranging from 10to 25%. About 30% of young people have a sleeping problem, and it is assumed to be the second most common sign of mental disorder [6]. Commercially available medications for treating anxiety have multiple side effects, exhibit dependence and tolerance on chronic treatment and have a still disappointing magnitude of improvement [7]. Therefore, plant-based natural medication has become the interest of researchers during the last few years. Several studies reported the plants famed for their traditional medicinal uses were investigated for their psychotherapeutic potential in animal models.

Diarrhea is an infection associated with rapid and frequent defecation, lower fluid absorption, and enhanced gastrointestinal motility, which remained one of the world’s top causes of mortality till today. Even many civilized countries are experiencing a substantial percentage of the said disease risk. It has been listed as the second strongest cause of mortality in children younger than 5 years old, accounting for over 1.5 million deaths annually. Oxidative stress (OS) is also responsible for the pathogenesis of gastrointestinal tract (GIT) complications, including diarrhea [8]. Several synthetic drugs are available to treat diarrhea, including anti-secretory, anti-spasmodic, and prostaglandin inhibitors (i.e., loperamide and atropine), posing many adverse effects (severe constipation, abdominal pain, irregular heartbeat, severe dizziness) and drug interactions. The World Health Organization (WHO) has accentuated the use of natural medicines for the prevention and treatment of diarrheal diseases [9].

*Duabanga grandiflora* (DC.) Walp. (Family: Lythraceae) is a large evergreen tree and indigenous to Southeast Asia, Thailand, Myanmar, Nepal, Laos, Vietnam and, most prominently, in the forest and hill tracks of Bangladesh. The leaf extract of this plant has traditionally been used in the tribal areas for the management of joint pain, eye irritation, stomach ache, skin diseases, anti-inflammation, and anti-aging [10]. However, based on recent investigational findings, *Duabanga grandiflora* (DC.) Walp. leaf extract displayed a variety of pharmacological responses including, antioxidant, antibacterial, anti-diarrheal, antiviral, and antipyretic potential [11,12,13,14]. However, the present series of studies with the combination of experimental and computer-aided mechanisms were designed to scrutinize anxiolytic, anti-depressant, and anti-diarrheal potentials of the methanol extract of *D. grandiflora* stem bark extract using different experimental models.

## 2. Materials and Methods

### 2.1. Chemicals and Reagents

The methanol was purchased from the Sigma Chemical Company, St. Louis, MO, USA). Loperamide, fluoxetine hydrochloride and diazepam (standard drugs) were procured from Eskayef Pharmaceutical Ltd., Bangladesh. Normal saline solution (0.9% NaCl) was obtained from the Social Marketing Company (SMC) Ltd., Bangladesh, and Tween-80 was collected from BDH Chemicals (Leicestershire, UK). All other reagents of analytical grade were purchased from local sources through Taj Scientific Ltd., Chittagong, Bangladesh.

### 2.2. Plant Materials

The fresh stem barks of *D. grandiflora* were collected from SitaPahar, Kaptai, Rangamati district (Chittagong, Bangladesh) throughout the winter season of 2018. The specimen (no. DP-S0372) was collected and deposited at the Department of Pharmacy, International Islamic University Chittagong, Bangladesh for future reference. Collected stem barks were shade-dried at room temperature (25 ± 1 °C), and finely powdered using a mechanical grinder and stored in an airtight sealed container.

### 2.3. Extract Preparation

Approximately 400 g of coarse powder was dissolved in methanol (sample: solvent; 1:6) at room temperature (25 ± 2 °C) for 9 days with random shaking and stirring [15]. The supernatant was then filtered using a cotton plug followed by Whatman #1 filter paper. Finally, the filtrate was concentrated by evaporating the solvent using a rotary evaporator (Buchi R114, Postfach, Switzerland) at 40 °C under alleviate pressure to evaporate the solvent. The 46.50 g crude extract of MEDG was obtained (yield value, 11.625%) and preserved at 4 °C for subsequent experiments.

### 2.4. Phytochemical Observation

#### 2.4.1. Qualitative Phytochemical Analysis

The MEDG (1 g) was dissolved in 100 mL of methanol for preparing the stock solution. The stock solution was used for the preliminary qualitative phytochemical screening using the described method in [16] to scrutinize the presence of secondary metabolites specifically alkaloids, carbohydrates, flavonoids, saponins, tannins, glycosides, proteins, phenols, triterpenoids, and phytosterols.

#### 2.4.2. Qualitative Phytochemical Analysis

##### Determination of Total Phenolic Contents

The total phenolic content (TPC) was evaluated with Folin–Ciocalteu reagent (FCR) using the modified method described earlier [17].

##### Determination of Total Flavonoid Contents

The total flavonoid content (TFC) was determined by the aluminum colorimetric method, as reported earlier [8].

### 2.5. Study Animals

Male Swiss albino mice (6–7 weeks) weighing between 22 and 30 g were purchased from the animal research division of the Bangladesh Council of Scientific and Industrial Research (BCSIR), Chittagong, Bangladesh. The experimental animals were acclimatized for 14 days before the experiment. They were kept in laboratory animal cages and maintained in a standard laboratory environment (12 h dark/12 h light cycle; RH: (55–65%); RT: (25 ± 2 °C) with standard feed (ad libitum) and fresh water. Additionally, the mice were fasted overnight and weighed before starting an experiment.

### 2.6. Acute Toxicity Test

The experimental design was accomplished following the previously described procedure with slight modification [18]. The animals were divided into two groups (control and test), comprising six animals (*n* = 6) in each group. The control group received 1% tween 80 in saline water, while the test group received different doses of crude extract of MEDG (200 to 4000 mg/kg, p.o) [17]. The animals were monitored under close surveillance for the next 48 h and up to seven consecutive days to record any signs of an allergic reaction (itchy, rhinitis, red-eye, swollen lip, etc.), behavioral changes, and incidence of mortality.

### 2.7. Treatments

Experimental mice were divided into four treatment groups (Groups I–IV), where each group consists of six animals (*n* = 6). The negative control (Group I) received the vehicle (1% Tween 80 in DW, 10 mL/kg, p.o); the positive control (Group II) received the standard drug diazepam (1 mg/kg, b.w, i.p.) used for elevated plus-maze test and hole-board test; fluoxetine (20 mg/kg, b.w, i.p.) was used for the forced swimming test and tail suspension test; and loperamide (5 mg/kg, b.w, p.o) was used for castor oil-induced diarrhea, castor oil-induced enter pooling, and the charcoal-induced gastrointestinal motility test. The test groups (Group III and Group IV) received the MEDG extract at doses of 200 and 400 mg/kg (b.w, p.o), respectively. 

### 2.8. Anxiolytic Activity

#### 2.8.1. Elevated Plus Maze (EPM)

Anxiolytic behavior in mice was evaluated by using elevated plus maze (EPM) according to the modified method [19]. The apparatus is sketched with two open arms (35 × 5 cm), two closed arms (35 × 20 cm), and a central square (plus sign) (5 × 5 cm). The apparatus was placed at a height of 25 cm above the floor with an open ceiling. The experimental animals were treated randomly as per the experimental design indicated. After 30 min of administration, each mouse was placed in the central position with the head facing to one of the closed arms and observed for 6 min while the last 5 min was counted for anxiolytic behavior followed by adjustment for 1st min. During the observation period, the cumulative number of entries and time spent in the open arm was documented.

#### 2.8.2. Hole-Board Test

The hole-board test (HBT) test is a method widely used to evaluate anxiolytic activity. The test apparatus consists of a white wooden board (20 × 40 cm) with 16 equally distant holes and 15 cm in height above the floor. Mice were administered randomly as per the experimental design indicated. After 30 min of dose administration, mice were placed individually at the center of the board and observed for 6 min while the 1st min was kept for adjustment of the apparatus. The number of heads dipping into the holes was counted throughout the last 5 min trial period. The head dipping was recorded if both eyes faded into the hole [20].

### 2.9. Anti-Depressant Activity

#### 2.9.1. Forced Swimming Test

The forced swimming test (FST) test is the most popularly used biological animal model for evaluating anti-depressant activity [20,21]. In this experiment, mice were individually forced to swim 30 min before the test for a 6 min observation period into a transparent glass cylinder (height: 55 cm, diameter: 30 cm) filled with 25 cm (depth) of water and maintained at 25 ± 1 °C. The last 4 min of a 6 min observation period was recorded as latency and immobility time for each animal, while the first 2 min were kept for adaptation of the apparatus. Mice were treated randomly as per the experimental design indicated. Immobile time was appraised if the animal made no further strive to escape besides the requisite action to hold its head above the water.

#### 2.9.2. Tail Suspension Test

The total period of immobility was measured by TST as described [21,22] for evaluating depressive-like behavior. Mice were treated randomly as per the experimental design indicated. After 1 h of dose administration, mice were suspended in a wooden suspension box at the height of 50 cm above the basement by adhesive tape placed approximately 1 cm from the tip of the tail. During immobility, the last 4 min of a 6 min period were considered, while the first 2 min were kept for initial adjustment. If the mouse was hanging passively and motionless with its head straight to the bottom of the box, it was immobile. Decreasing immobility time during TST has an anti-depressant potential.

### 2.10. Anti-Diarrheal Activity

#### 2.10.1. Castor Oil-Induced Diarrhea

A castor oil-induced diarrheal study was performed by following the method described earlier [23]. The dosing of the randomly divided mice was conducted as per the experimental design indicated. After 1 h, all mice were treated with castor oil (0.5 mL/mouse, p.o.) and placed into separate cages consisting of non-wetting transparent paper. The parameters including the onset of diarrhea, the total feces, amount of wet feces, the weight of wet feces, and weight of all feces were counted every hour for a 4 h observation period. The percentage inhibition of defecation was calculated using the following formula:

% inhibition of defecation = [Mc − Mt/Mc] × 100

where Mc = mean number of wet feces in the control group and Mt = mean number of wet feces in the test group.

#### 2.10.2. Castor Oil-Induced Enter Pooling

Castor oil-induced enter pooling was performed by following the method described earlier with minor modifications [24]. The dosing of the randomly divided mice was conducted as per the experimental design indicated. Immediately afterward, each mouse was orally treated with castor oil (0.2 mL). After 30 min, mice were sacrificed, and the small intestine of each mouse was ligated both at the pyloric sphincter and the ileocecal junctions and dissected out. The intestinal contents were collected by milking into a graduated tube, and the volume was measured. The intestines were reweighed, and the difference between full and empty intestines was calculated.

#### 2.10.3. Gastrointestinal Motility Test

Charcoal-induced gastrointestinal motility was performed by following the method described in [24]. The dosing of the randomly divided mice was conducted as per the experimental design indicated. The experimental mice were given the drug orally 1 h before administering 1 mL of charcoal suspension (10% charcoal, 5% gum acacia). After 1 h, experimental animals were sacrificed, and the small intestine (from the pylorus to caecum) was removed. The total length of the small intestine was estimated using a calibrated autocrat. Hence, the total length of the intestine (distance travel by charcoal marker) was measured, and the percentage of inhibition was calculated using the following formula:

% inhibition of defecation= [Dc − Dt/Dc] × 100

where Dc = distance travel by the charcoal in the control group and Dt = distance travel by the charcoal in the test group.

### 2.11. In Silico Computer-Aided Molecular Investigation

#### 2.11.1. Ligand and Protein Preparation

Seven lead isolates of MEDG were selected based on a comprehensive literature study and a PASS analysis, namely duabanganal A, duabanganal B, duabanganal C, duabanganal D, vanillin, oleanolic acid, and betulinic acid [25] were uncovered from the PubChem database in SDF format [26]. The selected isolates are converted into a 3D format from 2D, and then, the atom energy was minimized using the LigPrep module (forced field OPLS 2005) of Schrodinger Maestro 2017. The three-dimensional structure of specified proteins was collected in PDB format from the Protein Data Bank. Potassium channel receptor (PDB id: 4UUJ) was assessed for anxiolytic activity, human serotonin transporter receptor (PDB id: 5I6X) was assessed for anti-depressant activity, and staphylcoccalnulease variant Delta+PHS receptor (PDB id: 4WRD) was assessed for anti-diarrheal activity. After that, Glide of Schrodinger Maestro (v11.1) was fixed and optimized using a protein preparation wizard [18,27].

#### 2.11.2. Grid Generation and Docking Analysis

The aim of the molecular docking studies was to establish the potential mode of action of seven specified isolates of MEDG against particular receptors. The Glide software was used to choose a versatile ligand docking system for this project. Glide percent has been used to calculate the final score, which was based on energy-saving gestures [17]. In order to ensure accuracy, each docking was run three times. Finally, the docked samples were extracted in SDF format for visualizing the docking interaction using the discovery studio visualizer (v3.0).

### 2.12. Ethical Considerations

All of the experiments using animals were conducted in the Department of Pharmacy, International Islamic University Chittagong, Bangladesh. ‘Principles of the Laboratory Animal Care’ and ‘National Animal Care Laws’ were strictly followed during the handling of animals for the study. The study protocol was approved by the Department of Pharmacy, International Islamic University Chittagong, Bangladesh (Ref.: IIUC/PHARM-AEC-52/10-19).

### 2.13. Statistical Analysis

The experimental data are represented as mean ± SEM, and all of the test groups (*n* = 6 in each group) were compared with the control group to evaluate the statistical differences. Statistical analysis was evaluated using one-way analysis of variance (ANOVA) followed by Dunnett’s test for anti-depressant, anxiolytic, and anti-diarrheal investigation using GraphPad Prism v8.0 (GraphPad Software Inc., San Diego, CA, USA). *p* values < 0.001, 0.01, and 0.05 were considered statistically significant.

## 3. Results

### 3.1. Phytochemical Analysis

#### 3.1.1. Qualitative Analysis

The qualitative phytochemical screening of MEDG showed the presence of alkaloids, flavonoids, saponins, tannins, phenols, triterpenoids, and phytosterols (Supplementary Table S1).

#### 3.1.2. Total Plant Phenolics and Flavonoids

Quantitative analyses revealed that the MEDG contained the highest amount of phenolic content (91.62 ± 0.78 mg of GAE/g of dried extract) and flavonoid content (68.81 ± 0.55 mg of QE/g of dried extract) (Table 1).

### 3.2. Acute Toxicity Test

During the closely monitored observation period, it was observed that the oral administration of MEDG doses up to 4000 mg/kg was well tolerated by the experimental animals. The results indicated that MEDG (200 and 400 mg/kg) exhibited a low toxicity profile, and these doses were considered for present experimental evaluation.

### 3.3. Effect of MEDG on Anxiolytic Activity

#### 3.3.1. Elevated Plus-Maze Test (EPM)

**Figure 1:**
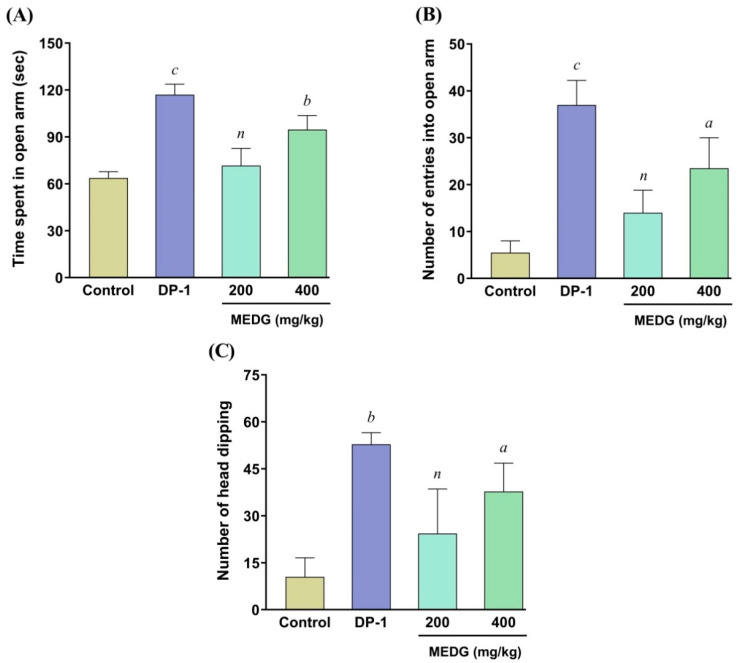
Effect of oral administration of MEDG on elevated plus maze test, time spent in open arm (**A**), the number of entries into open arm (**B**), and hole board test **(C)** in mice. The results were expressed as mean ± SEM, (*n* = 6), and ^a^ *p* < 0.05, ^b^ *p* < 0.01, and ^c^ *p* < 0.001 were statistically significant and (*n*) = non-significant compared with the control group followed by Dunnett’s test (one-way ANOVA). DP = diazepam; MEDG = methanol extract of *D. grandiflora* stem barks.

In the EPM test, the tested doses of MEDG showed increasing entries into the open arms and number of entries into the open arms (Figure 1A,B). MEDG (400 mg/kg) showed a statistically significant (*p* < 0.05, <0.01) rise in both times spent in open arms and the number of entries in open arms, respectively, in comparison with the control. The reference drug diazepam (1 mg/kg, i.p.) also significantly (*p* < 0.001) increased the time spent in the open arm and the number of entries into the open arm, respectively. Thus, the demonstrated anxiolytic activity on mice might be because of the stimulation of action of brain chemicals produced by the bioactive metabolites of the plant extract.

#### 3.3.2. Hole-Board Test (HBT)

As depicted in Figure 1C, the oral administration of MEDG revealed moderate effect at both doses (200 and 400 mg/kg), whereas the standard drug diazepam (1 mg/kg, i.p.) significantly (*p* < 0.01) increased the number of head dipping when compared with the vehicle-treated control group. Thus, the significant anxiolytic activity on experimental animals might be due to the stimulation of action of brain chemicals produced by the bioactive metabolites of the plant extract.

### 3.4. Effect of MEDG on Anti-Depressant Activity

**Figure 2:**
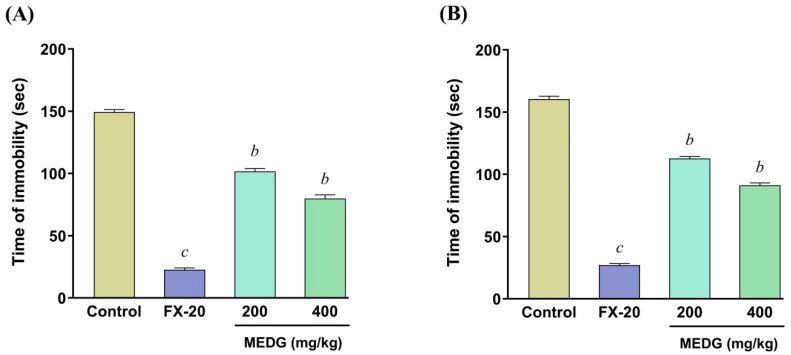
Effect of oral administration of the MEDG on the immobility time of forced swimming test (**A**) and tail suspension test (**B**) in mice. The results were expressed as mean ± SEM (*n* = 6), and ^b^ *p* < 0.01, and ^c^ *p* < 0.001 were statistically significant compared with the control group followed by Dunnett’s test (one-way ANOVA). FX-20 = fluoxetine HCl 20; MEDG = methanol extract of *D. grandiflora* stem barks.

The oral administration of MEDG on the duration of immobility time on both FST and TST is shown in Figure 2A,B. The findings revealed that, after oral administration of MEDG at the doses of 200 and 400 mg/kg and fluoxetine hydrochloride at a dose of 20 mg/kg, immobility time on both FST and TST showed significant (*p* < 0.01) depletion, respectively, compared with the control group. This anti-immobility effect on FST and TST might be due to the psychostimulant effect possibly produced by the secondary metabolites of the plant extract.

### 3.5. Effect of MEDG on Anti-Diarrheal Activity

#### 3.5.1. Castor Oil-Induced Diarrhea

The effects of the oral administration of MEDG on diarrheal inhibition of the castor oil-induced test are presented in Table 2. The current experimental findings revealed that the oral administration of MEDG (200 and 400 mg/kg) and loperamide (3 mg/kg) significantly (*p* < 0.001) delayed the onset of the duration of diarrhea and markedly reduced the total number of wet feces compared with the vehicle-treated control group. The extract showed 48.13% and 62.23% significant (*p* < 0.001) inhibition in defecation at the doses of 200 and 400 mg/kg where the standard drug displayed 88.58% diarrheal inhibition.

#### 3.5.2. Castor Oil-Induced Enter Pooling

The effect of oral administration of MEDG on castor oil induced-fluid accumulation is presented in Table 3. In this test, MEDG at doses of 200 and 400 mg/kg did not produce a significant and dose-dependent reduction in both the weight and volume of intestinal contents, whereas the reference drug loperamide (3 mg/kg) significantly (*p <* 0.01) exerted reductions in both the weight and volume of intestinal contents as compared with the vehicle-treated control group. The MEDG showed 53.22% and 66.13% inhibition on the weight of intestinal contents and 56.25% and 62.50% inhibition on the volume of intestinal contents at the doses of 200 and 400 mg/kg, whereas the reference drug loperamide (3 mg/kg) provided 70.96% and 75% inhibition, respectively.

#### 3.5.3. Charcoal-Induced Gastrointestinal Motility

The effect of MEDG on charcoal-induced gastrointestinal motility is summarized in Table 4. Compared with the vehicle-treated control group, MEDG (200 and 400 mg/kg) and loperamide (3 mg/kg) significantly (*p <* 0.001) decreased the small intestinal transit of charcoal meal in the experimental animals. MEDG (200 and 400 mg/kg) exhibited 61.35% and 75.25% inhibition of intestinal motility, respectively, while reference drug loperamide showed 82.96% compared with the control.

### 3.6. Molecular Docking Analysis

**Figure 3:**
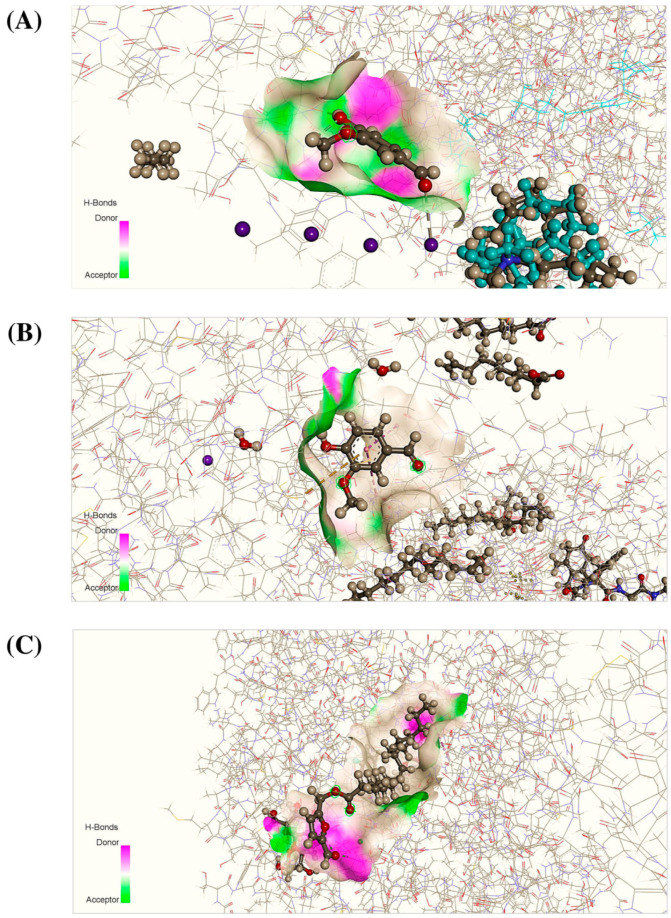
Three-dimensional binding interaction of vanillin with active site of 4UUJ (**A**), 4WRD (**B**), and duabanganal C with an active site of 5I6X (**C**), respectively, for anxiolytic, antidiarrheal and antidepressant potential.

Seven bioactive isolates of *D. grandiflora* (duabanganal A, duabanganal B, duabanganal C, duabanganal D, vanillin, oleanolic acid, and betulinic acid) were selected for the current in silico investigation. Based on an extensive literature review and a PASS prediction, these isolates provide very good Pa values for pharmacological activities such as anxiolytic, anti-depressant, and anti-diarrheal activities. For this reason, we selected these seven compounds for the in silico study. In addition, few studies reported that these compounds are most abundant among all the compounds present in the bark extract of this plant. These isolates were docked with numerous receptors, namely potassium channel receptor (PDB id: 4UUJ) for anxiolytic potential, human serotonin transporter receptor (PDB id: 5I6X) for anti-depressant potential, and staphylcoccalnulease variant Delta+PHS receptor (PDB id: 4WRD) for anti-diarrheal potential. Moreover, according to docking results, vanillin showed the highest docking scores (−4.60 and −5.04 Kcal/moL^−1^) against the 4UUJ and 4WRD receptors, respectively, wherein the docking scores of reference drugs diazepam and loperamide showed −4.37 and −5.15 Kcal/moL^−1^, respectively. Notably, in the case of anxiolytic potential, vanillin displayed a higher docking score than the reference drug diazepam. In addition, among the seven docked isolates, duabanganal C exhibited the highest binding score (−4.65 Kcal/moL^−1^) while the reference drug fluoxetine HCl displayed the best score (−6.72 Kcal/molL^−1^). Finally, these findings suggest that, out of all of the bioactive isolates investigated, vanillin exhibited a promising docking score for both anxiolytic and anti-diarrheal potential and duabanganal C exhibited promise for anti-depressant activity. The result of the binding affinities of selected compounds is reported in Table 5, and the best binding interactions are displayed in Figure 3. The present molecular docking study also exhibited different bond interactions alongside the docking score. Interestingly, vanillin demonstrated two hydrogen bonds and seven van der wall interactions with 4UUJ and two hydrogen bonds and five van der wall interactions with 4WRD, whereas diazepam demonstrated eight van der walls and one hydrogen bond interaction with 4UUJ and loperamide demonstrated eight van der wall interaction and two hydrogen bond interaction with 4WRD for anxiolytic and anti-diarrheal potential, respectively. In terms of anti-depressant activity, duabanganal C had three hydrogen bond interactions and eleven van der wall interactions, whereas the reference drug had four hydrogen bond interactions and nine van der wall interactions. Overall, the results show that only a few of the seven isolates have a strong binding interaction and a lower docking score than the reference drug.

## 4. Discussion

Natural products are a robust resource for medicine, as there are a variety of chemical constituents with strong pharmacological properties. Surprisingly, since ancient times, medicinal herbs have provided readily accessible, inexpensive, and effective medication. Various ethnomedicinal plants have been explored for their multiple biological effects and now serve as a choice in modern medicine [10,28]. Owing to its diverse group of bioactive compounds, *D. grandiflora* has the potential to produce many biological activities. Based on the recent antimicrobial activity, MIC analysis revealed that the crude ethyl acetate extract of this plant has antimicrobial properties against MRSA and MSSA. The combination’s mechanisms of action on MRSA were discovered to be linked to PBP2a inhibition, which is noteworthy [11]. The qualitative phytochemical screening in the current study confirmed the presence of alkaloids, flavonoids, saponins, tannins, phenols, triterpenoids, and phytosterols.

Anxiety and depression are considered the most prominent health concerns nowadays. The present study investigated anxiolytic activity using the elevated plus maze test (EPM) and hole board test (HBT). In the experimental analysis of EPM, the anxiolytic effect was observed by the elevation of entries and time spent in open arms, whereas an increase in entries and time spent in open arms was considered to show an anxiolytic effect [29]. MEDG revealed a significant anxiolytic effect indicated by enhanced open arm-entries and time spent in the open arm at a higher dose (400 mg/kg) compared with the control group. The HBT provided a tool for evaluating animal response to a traumatized environment that can assess anxiolytic behavior [30]. Notably, a disproportion of neurotransmitters of GABA-ergic pathways may cause anxiety. The anxiolytic potential was observed due to the opening of gamma amino butyric acid activating chloride channels that intensify the response of GABA receptors [31]. According to these findings, head-poking behavior is directly correlated with their emotional responses. In this study, MEDG provided no greater propensity of head dipping at any dose, which may be attributed to the absence of specific phytochemicals opening chloride receptors triggered by GABA as the anxiolytic effect relates specifically to GABA receptor activation.

The FST and TST are the most validated animal models used to evaluate anti-depressant activity. Imbalances in neurotransmitters such as GABA, serotonin, catecholamine, and noradrenaline have been implicated in the pathophysiology of depressive disorder [32]. Immobility generated in both FST and TST has been speculated to demonstrate the hopelessness and emotional complexity of animals. This study demonstrated a promising antidepressant-like effect in both FST and TST through a rapid decline in the duration of motionless time after the oral administration of the MEDG extract. MEDG doses decreased the time of immobility but did not have a calming effect in the mouse as the test mouse had adjusted to the environment prior to the experiment. Most interestingly, anti-depressant medication works through serotonin reuptake inhibition; hence, a similar mechanism of action could be predicted for the investigated plant extract. In addition, research studies reported that the normal function of anti-depressants regulated by stimulating noradrenaline and serotonergic transmission in the brain cells agreed with the apoptogenic effect of the plant extract.

Diarrhea results when there is an imbalance in active ion transport via increased luminal osmolarity or hydrostatic tissue pressure, enhanced Cl^−^ secretion or suppressed Na^+^/K^+^ absorption, and irregular intestinal motility [33]. In anti-diarrheal screening, castor oil incites diarrhea through a variety of mechanisms, namely, ricinoleic acid, which causes irritation and inflammation of the GI mucosa by binding to EP3 proteinoid receptors on smooth muscle cells, releasing several inflammatory mediators (histamine, NO, and tachykinins) that eventually stimulate intestinal motility and electrolyte discharge from the gut. It is reasonable to link the decrease in motor small intestine activity to defective NO production/release, which increases resistance to flow and decreases intestinal transit. In particular, experiments utilizing NOS inhibitors have shown that diminished nitrergic relaxation at the level of the small intestine results in delayed intestinal transit [34]. HECLP’s suppression of intraluminal fluid accumulation could be attributed to prostaglandin production inhibition. An increase in water and electrolyte reabsorption through the intestinal mucosa could potentially explain the decrease in intestinal porosity and permeability [35]. It has been outlined that the administration of castor oil enhanced malonaldehyde production in the GIT mucosa, indicating an augmentation of lipid peroxidation, which could be a possible mechanism of tissue alteration by oxygen-reactive derivatives [36]. In this study, MEDG demonstrated a significant inhibition in castor oil-induced diarrhea and inhibited the reduction in charcoal meal on charcoal-induced GI motility by decreasing the length of the peristalsis index compared with the negative control. In the castor oil-induced enter pooling method, MEDG showed no significant effect. According to the qualitative phytochemical data, the plant extract contains flavonoids, saponins, tannins, and triterpenoid, which could avert the alteration of hydro electrolytic secretions and intestinal motility. Hence, it is plausible to assume that these metabolites could be responsible for the inhibition of defecation and intestinal motility by the MEDG extract.

In addition to these experimental techniques, a computer-aided drug design is used to correlate the experimental results of the selected isolates/compounds of MEDG, to better explain the ligand–receptor complexes, and to see how the scientific evidence mirrored in silico findings. However, based on extensive literature review from previous studies, the current study selected a few isolates from thirty-eight identified isolates to correlate the experimental findings with the in silico molecular target. Notably, a previous study reported that major bioactive isolates, namely, 5-formylfurfuryl esters, duabanganals A–D, as well as sixteen known chemicals, a known 5-formylfurfuryl ester, latifolinal, eight pentacyclic triterpenes, a benzofuran derivative, an ellagic acid derivative, vanillin, b-sitosterol, b-sitosterol were identified. The structure of each identified compound was determined on the basis of spectroscopic analysis [25]. A computer-assisted drug design, in specific, plays a significant role in stimulating pharmaceutical development [21]. Consequently, in silico approaches are the most effective computational technique for studying structural molecular ligand–receptor interactions and generating new knowledge of appropriate biochemical mechanisms of the natural compounds [37]. Furthermore, the in silico molecular approach can clarify the possible targets and mechanisms that characterize a wide range of pharmacological activities. To better target the results of the current experimental findings, the molecular modeling study was performed to further explain the molecular mechanisms. Particularly, the docking method employed in this study provided useful insight into the biologically active isolates at the molecular and cellular level bindings to various protein targets that are known to play crucial roles in pharmacological pathways, including anxiolytic anti-depressant and anti-diarrheal cascades. In the present experimental design based on an in silico molecular docking analysis, specific target receptors (4UUJ, 5I6X, and 4WRD) were evaluated for the anxiolytic, anti-depressant, and anti-diarrheal potential of seven bioactive isolates of MEDG. Notably, vanillin exhibited a promising docking score among the seven isolates with both anxiolytic and anti-diarrheal activities compared with the reference drugs diazepam and loperamide, respectively. Analogously, in the case of anti-depressant activity, duabanganal C displayed a promising docking score, which is comparable with the docking score of reference drug fluoxetine HCl. The present in silico drug design inferred that vanillin could be a promising bioactive natural isolate for anxiolytic and anti-diarrheal activities and that duabanganal C could be promising for anti-depressant activity and, hence, is suggested for next-step QSAR, molecular dynamics, and homology modeling studies.

Overall, the present findings do not conclude the core mechanism of action, and hence, extensive mechanistic studies are needed to clarify the issue.

## 5. Conclusions

The present investigations revealed the scientific basis for the neuropharmacological and anti-diarrheal potential of the stem bark extract of *D. grandiflora* for the very first time. Significant neuropharmacological as well as antidiarrheal potential was observed with the MEDG treatment, which was reported to be comparable with the effects of the corresponding reference drugs. These pharmacological activities could be possibly attributed to the presence of a variety of secondary metabolites in the stem bark extract. Moreover, in the molecular docking analysis, several bioactive promising molecules exhibited optimistic binding affinity to specific proteins, which strongly supports the experimental data. Therefore, further investigations, followed by the isolation and characterization of lead metabolites and standardization of the different plant parts with varied sources are strongly recommended. In addition, their particular mechanistic investigations are also essential to justifying these present potential outcomes.

## Figures and Tables

**Table 1:** Analysis of total plant phenolics and total plant flavonoids of MEDG.

Phytochemical	MEDG	Regression Equation
Phenolics (mg GAE/g extract)	91.62 ± 0.78	y = 0.0039x + 0.033 R^2^ = 0.9987
Flavonoids (mg QE/g extract)	68.81 ± 0.55	y = 0.0102x − 0.0659 R^2^ = 0.9651

Values are represented in mean ± SEM (*n* = 3). MEDG = methanol extract of *D. grandiflora* stem barks.

**Table 2:** Effects of MEDG on castor oil-induced diarrhea in mice.

Treatment	Dose	Onset of Duration (min)	Number of Wet Feces	Total of Feces	% Inhibition of Defecation
Control (1% tween 80)	10 mL/kg	67.16 ± 1.05	10.16 ± 0.89	13.67 ± 0.80	-
Standard (Loperamide)	3 mg/kg	186.16 ± 2.03 ^c^	1.16 ± 0.56 ^c^	2.44 ± 0.77 ^c^	88.58
MEDG	200 mg/kg	132.16 ± 1.98 ^b^	5.27 ± 1.23 ^b^	7.16 ± 0.30 ^b^	48.13
	400 mg/kg	178.83 ± 1.64 ^c^	3.33 ± 1.10 ^c^	4.83 ± 0.60 ^c^	62.23

Values are represented in mean ± SEM (*n* = 6). One-way analysis of variance (ANOVA) followed by Dunnett’s test was employed, where ^b^ *p* < 0.01, and ^c^ *p* < 0.001 were statistically significant compared with the control. MEDG = methanol extract of *D. grandiflora* stem barks.

**Table 3:** Effects of MEDG on castor oil-induced enter pooling in mice.

Treatment	Dose	Weight of Intestinal Content (gm)	% of Inhibition	Volume of Intestinal Content (mL)	% of Inhibition
Control (1% tween 80)	10 mL/kg	0.62 ± 0.02	-	0.48 ± 0.02	-
Standard (Loperamide)	3 mg/kg	0.18 ± 0.89 ^b^	70.96	0.12 ± 0.71 ^b^	75
MEDG	200 mg/kg	0.29 ± 0.71 *^#^*	53.22	0.21 ± 0.80 ^a^	56.25
	400 mg/kg	0.21 ± 0.92 ^a^	66.13	0.18 ± 0.83 ^a^	62.50

Values are represented in mean ± SEM (*n* = 6). One-way analysis of variance (ANOVA) followed by Dunnett’s test was employed, where ^a^ *p* < 0.05, ^b^ *p* < 0.01, and (^#^) = non-significant were statistically significant compared with the control. MEDG = methanol extract of *D. grandiflora* stem barks.

**Table 4:** Effects of MEDG on charcoal-induced gastrointestinal motility in mice.

Treatment	Dose	Total Intestinal Length (cm)	Distance Moved by the Charcoal Meal (cm)	Peristaltic Index (%)	% of Inhibition
Control (1% tween 80)	10 mL/kg	51.22 ± 2.02	39.37 ± 1.80	76.86	-
RSD (Loperamide)	3 mg/kg	48.17 ± 0.67 ^c^	6.31 ± 0.77 ^c^	13.09	82.96
MEDG	200 mg/kg	50.55 ± 1.40 ^c^	15.02 ± 1.27 ^c^	29.71	61.35
MEDG	400 mg/kg	51.17 ± 1.49 ^c^	9.73 ± 0.64 ^c^	19.02	75.25

Values are represented in mean ± SEM (*n* = 6). One-way analysis of variance (ANOVA) followed by Dunnett’s test was employed, where ^c^ *p* < 0.001 were statistically significant compared with the control. MEDG = methanol extract of *D. grandiflora* stem barks.

**Table 5:** Docking score (kcal/moL) of the specified bioactive isolates for anxiolytic, antidepressant, and antidiarrheal potential.

Proteins	PDB ID: 4UUJ (Anxiolytic)	PDB ID: 5I6X (Antidepressant)	PDB ID: 4WRD (Antidiarrheal)
Ligands	Docking Score	Docking Score	Docking Score
Diazepam	−4.37	-	-
Fluoxetine	-	−6.72	-
Loperamide			−5.15
Duabanganal A	−4.44	−4.57	−4.65
Duabanganal B	−3.38	−4.54	−4.62
Duabanganal C	−3.47	−***4.65***	−3.69
Duabanganal D	−3.27	−3.69	−3.24
Vanillin	−***4.60***	−4.13	−***5.04***
Oleanolic acid	−2.19	−3.00	−4.50
Betulinic acid	−1.84	−2.43	−3.32

The best binding interactions score is shown by bold italic values.

## Data Availability

The data that support the findings of this study are available on request.

## Supplementary Materials

The following supporting information can be downloaded at https://www.mdpi.com/article/10.3390/cimb44050159/s1, Table S1: Phytochemical Screening of MEDG.
