# Supplementary material for: Neuropharmacological and Antidiarrheal Potentials of Duabanga grandiflora (DC.) Walp. Stem Bark and Prospective Ligand–Receptor Interactions of Its Bioactive Lead Molecules
Source: Curr Issues Mol Biol. 2022 May 20;44(5):2335–49. doi: 10.3390/cimb44050159 (PMC9164075; doi:10.3390/cimb44050159)
Supplement: Supplementary file 1 [file cimb-44-00159-s001.zip › cimb-1662014-supplementary.pdf]

Supplementary materials

Table S1. Phytochemical Screening of MEDG

Phytochemicals	Name of the test	Observation
Alkaloid	Meyers Test	++
	Wagners Test	++
Carbohydrate	Molisch Test	-
	Benedict Test	-
Flavonoids	Hydrochloric acid Test	+++
Saponins	Foam Test	++
Tannins	Potassium dichromate Test	+
Glycoside	Acetic acid test	-
Protein	BSA Test	-
Phenol	Ferric chloride Test	++
Triterpenoids	Acetic anhydride Test	+
Phytosterol	Salkowski's Test	++
	Libermann Burchard's Test	+

Here, '+'= Present; '++'= Abundantly present; '-'= Absent; MEDG = methanol extract of *D. grandiflora* stem barks.
